# Recent developments in typhoidal and non-typhoidal *Salmonella* vaccines

**DOI:** 10.1097/QCO.0000000000001230

**Published:** 2026-08-05

**Authors:** Michael Blank, Anna Rydlova, Malick M. Gibani

**Affiliations:** Department of Infectious Disease, Imperial College London, London, UK

**Keywords:** enteric fever, immunological correlates, non-typhoidal *Salmonella*, *Salmonella*, vaccine development, vaccines

## Abstract

**Purpose of review:**

Typhoidal and invasive non-typhoidal *Salmonella* (iNTS) serovars remain leading causes of bloodstream infection, mortality and morbidity, particularly in South Asia and sub-Saharan Africa. The rising burden of antimicrobial resistance has strengthened the case for vaccination as a control strategy. This review summarises recent advances in *Salmonella* vaccine development, highlighting progress, remaining challenges and future directions.

**Recent findings:**

The introduction of typhoid conjugate vaccine (TCV) programmes across some endemic countries represents a major public health milestone. Important questions remain, however. These include the durability of protection, optimal booster strategies and performance in diverse epidemiological settings. Vaccine development for *S.* Paratyphi A and iNTS has also accelerated, with candidates including conjugate, outer membrane vesicle/GMMA and live-attenuated, platforms advancing through preclinical and early clinical evaluation. Multivalent vaccine strategies have attracted growing interest. However, the divergent epidemiology of typhoidal and non-typhoidal disease, including differences in age-specific risk and geographic distribution, raises important questions about whether a single combination product is suitable for all settings.

**Summary:**

The *Salmonella* vaccine landscape is evolving rapidly from a focus on typhoid alone toward broader protection against multiple clinically important serovars. Although recent progress is encouraging, important scientific, regulatory, and implementation challenges remain. The practical case for integrated *Salmonella* vaccine strategies will depend on careful matching of vaccine composition to the differing epidemiology, age-specific risks, and delivery contexts of typhoidal and nontyphoidal disease.

## BACKGROUND

*Salmonella* infection remains a significant global health priority, encompassing a broad spectrum of disease, ranging from self-limiting gastrointestinal illness to life-threatening invasive bloodstream infection [1]. The most common clinical presentation of human *Salmonella* infection is foodborne gastroenteritis, affecting an estimated 95.1 million people worldwide each year [2]. Beyond enterocolitis, typhoidal and non-typhoidal *Salmonella enterica* (NTS) serovars collectively rank among the leading causes of community-onset bloodstream infection in sub-Saharan Africa and parts of Asia [3^▪^,^4^,^5^].

**FIGURE 1:**
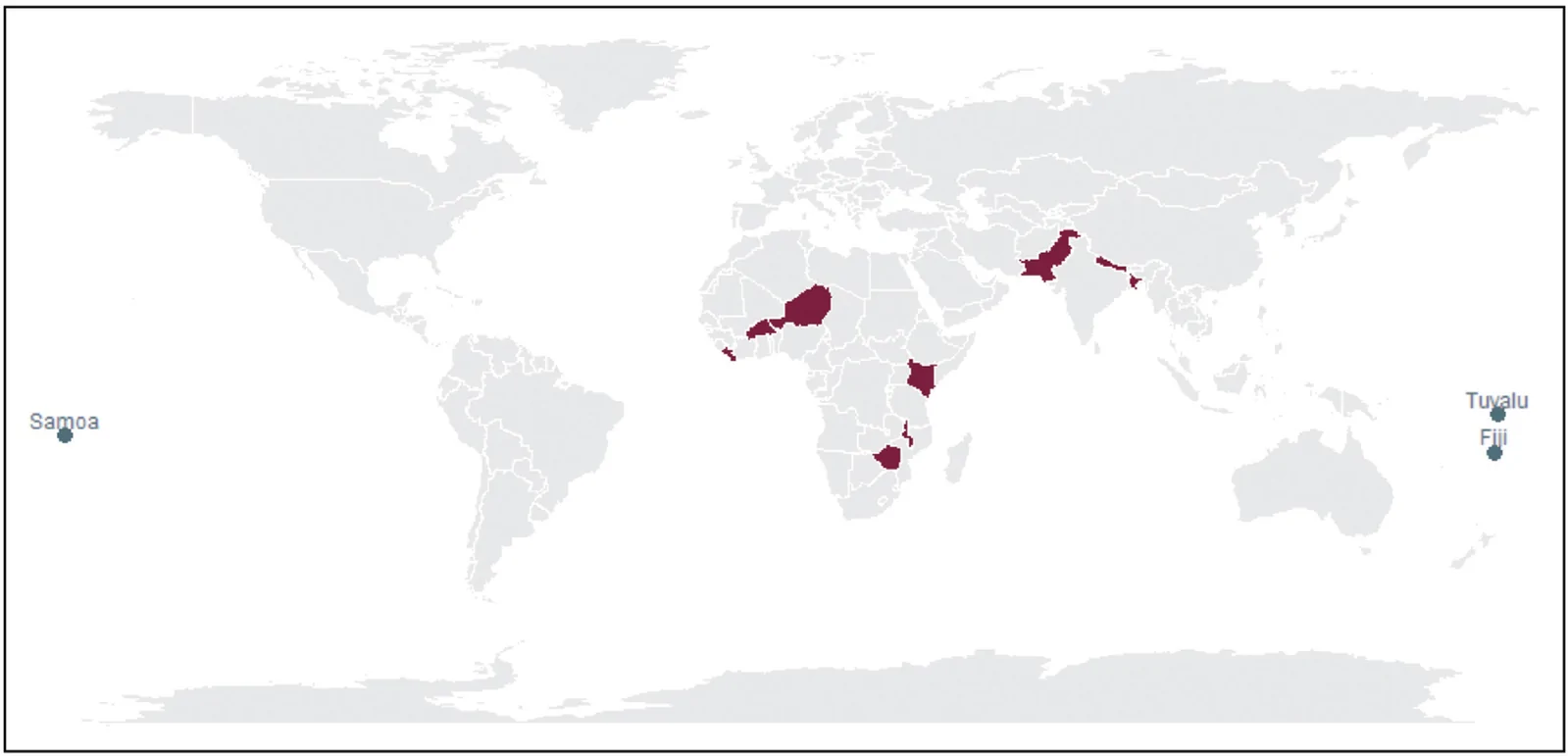
Countries with typhoid conjugate vaccine (TCV) implementation to date. The map shows countries that have introduced TCV through national routine immunisation programmes and/or nationally endorsed vaccination campaigns, including catch-up, preventive, and mass vaccination activities. South Pacific countries are labelled because of their small geographic size. Countries shown: Asia: Bangladesh, Nepal, Pakistan; Africa: Burkina Faso, Kenya, Liberia, Malawi, Niger, Zimbabwe; South Pacific: Fiji, Samoa, Tuvalu.

The past decade has seen striking advances in *Salmonella* vaccine development, most notably the emergence of new-generation Vi-polysaccharide-conjugate vaccines against typhoid. The advent of typhoid conjugate vaccines (TCVs) is particularly timely given the rising rates of antimicrobial resistance (AMR) amongst *Salmonella*[6]. Efficacious TCVs, which are WHO prequalified, arguably represent one of the most tangible public health advances of the past decade [7]. In particular, they are an exemplar of vaccines against AMR-associated pathogens. Figure 1 shows the 12 countries who have national TCV roll-out programmes to date. 

**Box 1:**
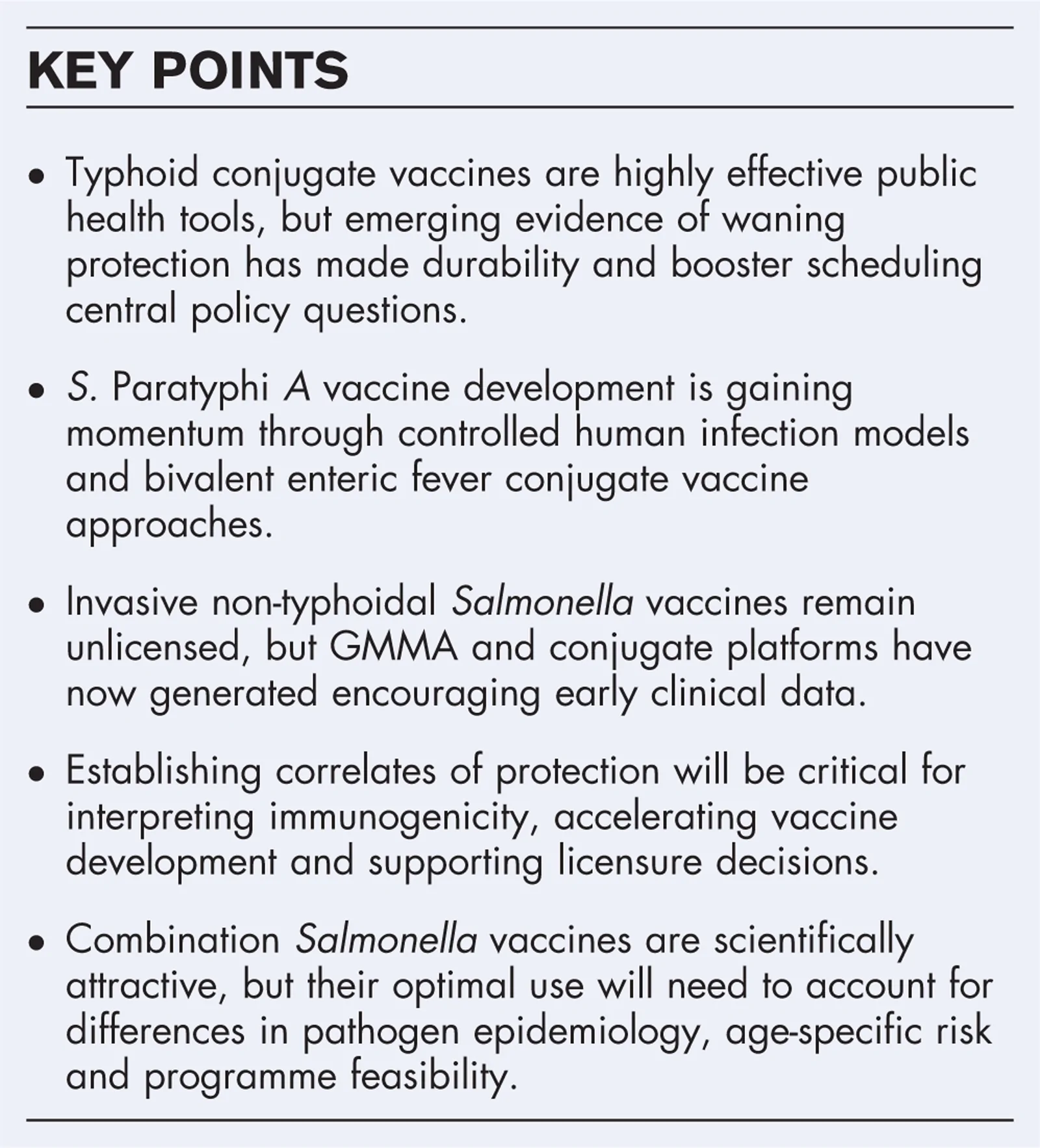
no caption available

Despite marked advances, challenges remain. Evidence of waning immunity following a single dose of TCV raises questions about the durability of protection, particularly in children vaccinated before the age of two, in whom postvaccine immune responses appear to decline most rapidly. This observation has brought the timing of vaccination and the need for booster doses into focus. Determining the optimal age of primary vaccination, and how this fits within existing “Expanded Programme on Immunisation” schedules (without disrupting established delivery platforms) remains an active area of debate [8]. Uptake remains a challenge, driven in varying degrees by vaccine hesitancy, health system capacity, and the need for community trust, which are all well recognised barriers to achieving the coverage levels needed for population-level benefits.

Beyond typhoid, vaccines against *Salmonella* Paratyphi A and the commonest serovars responsible for invasive NTS (iNTS, specifically *Salmonella* Typhimurium and *Salmonella* Enteritidis), remain at an earlier stage of development, but early data show promising safety and immunogenicity. The prospect of combination vaccines that span Typhi, Paratyphi A, and iNTS is appealing as a means of reducing the infection burden. However, the divergent epidemiology of enteric fever and iNTS – differing in geography, age distribution, and at-risk populations – raises genuine questions about whether a *Salmonella* combination vaccine strategy is epidemiologically and logistically coherent in all settings. Finally, these scientific and operational challenges are evolving in a difficult landscape, with looming drops in funding and headwinds for global vaccine development. Since Gavi support has been central to TCV introduction in eligible countries, sustainable procurement for LMICs will require long-term planning that extends beyond individual product approval.

This review summarises recent advances in vaccines for enteric fever and iNTS disease and discusses the emerging programmatic challenges of scale-up. We consider the key questions and future directions that will shape *Salmonella* vaccine policy over the coming decade.

## S. TYPHI

The 2018 WHO recommendation for countries with a high incidence of typhoid or significant antimicrobial resistance levels is for a single dose of TCV to be given from 6 months of age, with administration often aligned with measles-containing vaccine visits. Since then, several countries have introduced TCV into routine immunisation schedules, with an estimated 150 million children vaccinated by 2026 [9]. However, the same recommendations highlighted an important evidence gap regarding whether protection after a single dose would be sustained into later childhood, when typhoid risk often remains high [7].

WHO-prequalified TCVs, alongside candidates in development, are summarised in Table 1. A diversified vaccine pipeline remains important for ensuring access, supply security, and market stability. A new Vi-CRM197 conjugate vaccine has recently shown favourable results compared with the licensed Typbar TCV, although it did not meet non-inferiority at 6 months when anti-Vi IgG titre was used as the outcome [10]. Seroconversion nevertheless remained high, and longer-term follow-up will be important for assessing the durability of immune responses.

**Table 1:** Typhoid conjugate vaccines and vaccine candidates targeting Salmonella Typhi and *S.* Paratyphi A

Name (developer)	Subunit target	Population	Schedule or stage of development
*Licensed typhoid conjugate vaccines*			
Typbar-TCV (Bharat Biotech)	Vi-TT	6 months to 65 years	1 dose
TYPHIBEV (Biological E)	Vi-CRM197	6 months to 45 years	1 dose
SKYTyphoid (SK bioscience)	Vi-DT	6 months to 45 years	1 dose
ZyVac TCV (Zydus Lifesciences)	Vi-TT	6 months to 65 years	1 dose
Bio-TCV (Bio Farma)	Vi-DT	9 months to 45 years	1 dose (not yet WHO prequalified)
*Vaccine candidates in preclinical or clinical development*			
EuTYPH-C (EuBiologics)	Vi-CRM197	6 months to 45 years (S. Typhi)	Phase 3 completed [43^▪^]
Typhoid Vi conjugate (Lanzhou Institute of Biological Products)	Vi-rEPA	2 to 5 years	Phase 2 [44]
HD-MAP, needle-free (Vaxxas / SK bioscience)	Vi-DT	n/a	Preclinical [45]
CVD 1902 (Center for Vaccine Development, University of Maryland / Bharat Biotech)	Attenuated *S*. Paratyphi A	*S*. Paratyphi A	Phase 2b [23^▪▪^]
SII-TCV(B) (Serum Institute of India)	Vi and O:2 conjugate	*S*. Typhi and *S*. Paratyphi A	Phase 2, BiVISTA trial [26]
Vi-CRM197 + O:2-CRM197 (GSK Vaccines Institute for Global Health / Biological E)	Vi-CRM197 and O:2-CRM197	*S*. Typhi and *S*. Paratyphi A	Phase 1 completed [24^▪^]

The upper section lists currently licensed typhoid conjugate vaccines; all are WHO prequalified with the exception of Bio-TCV, which is licensed in its country of manufacture but not yet prequalified. The lower section lists vaccine candidates in preclinical or clinical development against S. Typhi, S. Paratyphi A, or both. Developers or manufacturing partners are given in parentheses after the vaccine name. Subunit targets refer to the antigen and, where relevant, the carrier protein used in conjugation (TT, tetanus toxoid; CRM197, cross-reactive material 197; DT, diphtheria toxoid; rEPA, recombinant exoprotein A of Pseudomonas aeruginosa). Clinical trial registration numbers are given in parentheses where available.

Phase 3 studies in Malawi, Bangladesh and Nepal have established the protective efficacy of TCV, but longer-term follow-up has shown important variation in efficacy by setting and age at vaccination. In Malawi, TCV efficacy was 78.3% at 4.6 years (95% CI 66.3–86.1), with the lowest efficacy observed in children vaccinated between 9 months and 2 years of age (70.6%, 95% CI 6.4–93.0) [11^▪▪^]. In Nepal, TCV efficacy at 2 years was 79.0% (95% CI 61.9–88.5), though this study was underpowered to assess vaccine efficacy in the under 2 age group [12]. In Bangladesh, vaccine effectiveness fell from 85% at 17–18 months follow-up to 50% (95% CI −13 to 78) at 3–5 years. The decline was most marked in children vaccinated under 2 years of age, in whom efficacy at 5 years was 24% (95% CI −29 to 55) [13^▪▪^]. Taken together, these studies show that TCV provides substantial protection, but that the durability of protection is heterogeneous and may be lower when vaccination is given at younger ages [11^▪▪^,^13^^▪▪^,^14^,^15^].

Postmarketing surveillance has also provided reassuring evidence that TCV is safe and effective [19]. Real-world studies have confirmed that TCV reduces typhoid incidence, with a recent meta-analysis estimating vaccine effectiveness at 87% (95% CI 57–94%), with higher effectiveness among children older than 5 years [16^▪^]. Test-negative designs based on blood culture confirmation are particularly valuable in this setting, as they help control for biases linked to differential healthcare-seeking behaviour and case ascertainment. A posthoc test-negative analysis nested within the Malawi TCV trial, for example, yielded effectiveness estimates closely aligned with those of the parent randomised controlled trial [17].

Concern around waning immunity after a single dose remains a major consideration for TCV policy, particularly when vaccination is delivered in infancy. Immunogenicity studies show declining antibody concentrations over time, with reductions particularly marked in children vaccinated at younger ages [12,18,19]. However, the best correlate of protection remains uncertain, making it difficult to infer clinical protection from antibody kinetics alone. The 5-year follow-up study in Bangladesh suggested a three-fold rise in typhoid fever incidence approximately 3–5 years after vaccination [13^▪▪^], supporting the concern that waning protection may become clinically important in some settings. Evidence for booster dosing remains variable, but recent studies suggest that booster doses can improve antibody responses, especially when longer intervals are used [15,20]. The key policy question is therefore whether the additional protection provided by a booster is sufficient to justify the logistical complexity of introducing a preschool or school-entry dose.

To better understand these trade-offs, the WHO has commissioned modelling studies comparing different dosing schedules and their public health impact. In these models, TCV at 9 months, delivered with a catch-up campaign for older children and followed by a booster at 5 years, reduced cases by a median of 48–64% and was associated with economic benefit, although the magnitude of benefit varied according to incidence and assumptions about waning immunity [21]. These modelling studies reinforce that the value of booster dosing will depend on local epidemiology, the durability of protection after infant vaccination and the feasibility of delivering additional doses at scale

## *S.* PARATYPHI *A*

*Salmonella* Paratyphi A shares important epidemiological features with *S.* Typhi, with the highest burden described in South Asia. Despite causing an estimated two million cases of enteric fever each year [22], no vaccine against *S.* Paratyphi A has yet been licensed. This reflects a historical focus on typhoid vaccines but also the challenge of demonstrating field efficacy for paratyphoid vaccines. In large phase 3 studies of TCV, for example, paratyphoid endpoints were relatively infrequent [13^▪▪^], highlighting the difficulty of using traditional efficacy trials for pathogens with lower or more heterogeneous incidence.

These challenges mean that the development and approval pathway for *S.* Paratyphi A vaccines is likely to differ from that used for TCVs. Controlled human infection models offer one approach to obtaining early efficacy readouts while also identifying putative correlates of protection. In a recent placebo-controlled CHIM study, an oral live attenuated *S.* Paratyphi A vaccine was safe and showed an efficacy of 73% (95% CI 46–86) following challenge, alongside induction of IgG and IgA responses to the *S.* Paratyphi A O:2 antigen [23^▪▪^].

Conjugate vaccines are also in development, including bivalent formulations combining Vi antigen with the *S.* Paratyphi A O:2 antigen. Phase 1 data from one such candidate showed an acceptable safety profile and induction of anti-Vi and anti-O:2 IgG responses, although a second dose did not clearly boost immune responses [24^▪^]. A phase 2 CHIM trial, BiVISTA, is now evaluating a bivalent *S.* Typhi/*S.* Paratyphi A conjugate vaccine, with paratyphoid challenge efficacy and serum anti-Vi IgG included as key outcomes [25^▪^]. If successful, this approach could support WHO-endorsed CHIM-based pathways to licensure [26].

Taken together, these studies suggest that the most promising pathway for *S.* Paratyphi A vaccine development may be through bivalent enteric fever vaccines supported by safety, immunogenicity, and CHIM efficacy data, rather than conventional field efficacy trials alone.

## NON-TYPHOIDAL SALMONELLA

To date, no vaccine against invasive non-typhoidal *Salmonella* disease has reached licensure. This remains an important gap particularly in sub-Saharan Africa, where invasive disease is driven predominantly by *Salmonella enterica* serovars Typhimurium and Enteritidis and causes substantial morbidity and mortality in young children and immunocompromised adults. Modelling suggests that an iNTS vaccine with 85–95% efficacy, delivered in infancy with a later dose at 9 months, could prevent an estimated 2.6 million cases over 10 years in sub-Saharan Africa [27^▪^], although the impact will depend on local incidence, vaccine coverage, demography, and durability of protection.

The primary target for most vaccines in development is the O-antigen, although protective antigens and immune correlates remain incompletely defined. Several candidates are in development, from preclinical platforms through to phase 2 studies in high-incidence settings, with controlled human infection models in development to support correlate identification (Table 2). GMMA vaccines present multiple surface antigens, may have intrinsic adjuvant activity, and incorporate lipid A modifications that reduce reactogenicity. A first-in-human dose-escalation study of an NTS GMMA vaccine showed acceptable safety and induced strong O-antigen immunoglobulin G (IgG) responses against *S.* Enteritidis O:9 and *S.* Typhimurium O:4,5, with corresponding serum bactericidal activity [28^▪^]. Trivalent conjugate vaccines are also progressing, including candidates combining *S.* Typhi Vi with core and O-polysaccharides from *S.* Typhimurium and *S.* Enteritidis. Early-phase studies have shown reassuring safety and immunogenicity results, supporting progression to a phase 2 adaptive dose-finding study in Malawi [29^▪^,^30^,^31^].

**Table 2:** Vaccine candidates in development for invasive non-typhoidal Salmonella (iNTS) disease, including combination products that also cover Salmonella Typhi or *S.* Paratyphi A

Name/description	Developer/manufacturer	Platform (valency)	Stage of development	Serovar coverage
iNTS-GMMA	GSK Vaccines Institute for Global Health (GVGH)	GMMA (bivalent)	Phase 2a (NCT06213506)	*S.* Typhimurium, *S.* Enteritidis
iNTS-GMMA + TCV	GSK Vaccines Institute for Global Health (GVGH)	GMMA + glycoconjugate (trivalent)	Phase 2a (NCT07286370)	*S.* Typhi, *S.* Typhimurium, *S.* Enteritidis
TCV + O:4 and O:9 glycoconjugate	Center for Vaccine Development, University of Maryland / Bharat Biotech	Glycoconjugate (trivalent)	Phase 2 (NCT05784701)	*S.* Typhi, *S.* Typhimurium, *S.* Enteritidis
Vi-DT + O:4 + O:9	International Vaccine Institute / SK bioscience	Glycoconjugate (trivalent)	Preclinical [46]	*S.* Typhi, *S.* Typhimurium, *S.* Enteritidis
O:4 + O:9+ SseB	Boston Children's Hospital / GSK	MAPS (bivalent)	Preclinical [38]	*S.* Typhimurium, *S.* Enteritidis
Vi + O:2 +O:4 + O:9 + SseB	Boston Children's Hospital / GSK	MAPS (quadrivalent)	Preclinical [47]	*S.* Typhi, *S.* Paratyphi A, *S.* Typhimurium, *S.* Enteritidis

All candidates target S. Typhimurium and S. Enteritidis, the two serovars responsible for the majority of iNTS disease globally. Selected combination products extend coverage to S. Typhi (trivalent formulations) or to S. Typhi and S. Paratyphi A (quadrivalent formulation). Platforms include generalised modules for membrane antigens (GMMA, outer membrane vesicle-based preparations), glycoconjugate vaccines comprising O-antigen polysaccharide linked to a protein carrier, and the Multiple Antigen-Presenting System (MAPS), in which polysaccharide and protein antigens are coupled non-covalently to a common scaffold. O-antigen specificities are O:2 for S. Paratyphi, O:4 for S. Typhimurium and O:9 for S. Enteritidis. SseB is a Salmonella-specific secreted protein incorporated into the MAPS constructs.

Establishing immunological correlates of protection remains a key bottleneck for iNTS vaccine development. Anti-O-antigen IgG and serum bactericidal activity are the leading binding/functional candidate correlates but lack a defined threshold for clinical protection [32,33]. A plausible route forward is to triangulate evidence across natural cohort studies, early-stage vaccine studies and CHIM, supported by standardised immune-assays and serum standards. A recently established S. Typhimurium CHIM (the CHANTS study) has completed recruitment and aims to identify immune responses associated with protection [34], while an upcoming re-challenge study (ISRCTN69516953) will assess infection-derived immunity.

A Full Value of Vaccines Assessment has highlighted the importance of aligning iNTS vaccine development with policy, financing, and implementation considerations [35^▪^,^36^,^37^]. Additional platforms, including multiple antigen-presenting system vaccines, reverse vaccinology, immunoinformatics, and machine-learning-supported antigen discovery, may broaden the future pipeline, although most remain preclinical for iNTS [35,38–40^▪^].

## FUTURE HORIZONS

The future priorities for typhoid vaccines have gradually shifted. Several efficacy and effectiveness studies have proven adequate short-term efficacy, but the next decade will be defined by key programmatic questions. These include – but are not limited to – where TCVs sit within already crowded infant schedules, when/if to offer booster doses, maintaining adequate coverage and ensuring sustainable political/donor commitment after initial Gavi support tapers. All will require sustained investment at a moment when the global vaccine funding environment is increasingly challenging.

For paratyphoid, there is growing momentum. O-antigen conjugate candidates have completed phase 1 evaluation [24^▪^], and the BiVISTA phase 2 CHIM is currently testing a bivalent Vi/O:2 conjugate against paratyphoid challenge, with results expected in 2026 and a further bivalent conjugate CHIM in planning [25^▪^]. Alongside the recent CHIM efficacy data for the oral live-attenuated CVD 1902 vaccine [23^▪▪^], multiple prelicensure efficacy readouts for paratyphoid vaccines will become available over the next 2−3 years. Because conventional phase 3 efficacy trials are widely considered infeasible given the heterogeneous epidemiology and lower incidence of paratyphoid, CHIM-based efficacy data, supplemented by well defined correlates and postlicensure effectiveness studies in endemic settings, are likely to remain the principal evidence pathway to licensure and deployment [26].

For iNTS, several vaccine candidates have generated encouraging safety and immunogenicity data but the field is approaching an inflection point. Translating this momentum requires a better-defined pathway towards generating efficacy or effectiveness data.

For combination products, geography and age distribution together determine where multivalent strategies offer real value. A quadrivalent vaccine covering Typhi, Paratyphi A, Typhimurium and Enteritidis is most plausibly attractive where typhoidal and iNTS burdens overlap significantly, as in parts of sub-Saharan Africa. The case is weaker where one disease dominates. In South Asia, enteric fever predominates, and adding iNTS components augments cost and complexity without proportionate benefit. Conversely, in many African settings, Paratyphi A remains uncommon and inclusion of an O:2 component delivers limited additional protection. Combination products should therefore be matched to setting rather than positioned as a single product fit for universal deployment.

Even where burdens overlap, the differing age distributions of typhoidal and non-typhoidal disease create a programmatic tension that has yet to be adequately addressed. Invasive NTS disease in sub-Saharan Africa peaks in infancy and early childhood, while typhoid risk is highest in school-age children [13^▪▪^]. A combination vaccine deployed early enough to maximise iNTS protection may therefore deliver weakening typhoid protection by the school years, when typhoid risk is greatest. This trade-off can in principle be addressed with a school-age booster, but doing so increases programmatic complexity, presupposes that the iNTS components themselves can be boosted effectively, and reintroduces implementation hurdles that single-dose TCV programmes were designed to avoid.

Translating that momentum into licensure and deployment will require a surveillance evidence base that does not yet exist. Sustained investment in multicountry surveillance was a key part of the pathway to TCV introduction, including the Strategic Typhoid Alliance across Africa and Asia (STRATAA), the Surveillance for Enteric Fever in Asia Project (SEAP), SEFI (Surveillance of Enteric Fever in India), the Typhoid Fever Surveillance in sub-Saharan Africa Program (TSAP), and the Severe Typhoid in Africa programme (SETA). Collectively, these generated the contemporary, age-stratified incidence data and standardised case ascertainment that aided planning of phase 3 trials and gave confidence to the 2018 recommendations [41]. An equivalent surveillance infrastructure for iNTS – generating standardised, age-stratified incidence estimates across high- and intermediate-burden settings in Africa and Asia – is now required before iNTS vaccine programmes can be rationally designed. Without these data, it will be difficult to define target populations, optimal age of vaccination and dosing schedules at the level of granularity that programmatic deployment requires.

Across all *Salmonella* serovars, progress would be accelerated by defining robust immunological correlates of protection. These remain poorly defined for invasive *Salmonella* disease and currently constrain rational comparison of candidates, dose selection, and interpretation of immunogenicity data [42].

## CONCLUSION

The *Salmonella* vaccine field has made substantial progress over the past decade. TCVs represent one of the clearest examples of public health vaccine progress in recent years and the paratyphoid and iNTS pipelines are now generating data that justify optimism. Real scientific challenges remain, including establishing immunological correlates of protection, understanding the durability of vaccine-induced immunity, and characterising the iNTS disease burden across Asia. Equally important are operational questions of sustaining TCV deployment, building surveillance infrastructure for iNTS programmes and creating combination products with appropriate rigour. Sustained attention to both will determine how much of that progress translates into lasting public health gain.

## Acknowledgements

*None.*

### Financial support and sponsorship

*This work received no specific external funding. M.B., A.R. and M.M.G. are supported by the NIHR Imperial Biomedical Research Centre. The views expressed are those of the authors and not necessarily those of the NIHR or the Department of Health and Social Care.*

### Conflicts of interest

*M.M.G. has received honoraria from GSK for nonpromotional speaking engagements. GSK is the parent company of the GSK Vaccines Institute for Global Health, which is developing several vaccine candidates discussed in this review. M.B. and A.R. declare no conflicts of interest.*

*AI disclosure: The authors used a large language model (Claude, Anthropic) for proofreading and basic copy-editing assistance during manuscript preparation. All content was reviewed and approved by the authors, who take full responsibility for the final manuscript.*`

## REFERENCES AND RECOMMENDED READING

Papers of particular interest, published within the annual period of review, have been highlighted as:

▪ of special interest
▪▪ of outstanding interest
